# Analysis of influencing factors of viral load in patients with high-risk human papillomavirus

**DOI:** 10.1186/s12985-020-01474-z

**Published:** 2021-01-06

**Authors:** Xuerong Lu, Tiantian Wang, Youzhong Zhang, Yuzhen Liu

**Affiliations:** 1 Department of Obstetrics and Gynaecology, Affiliated Hospital of Weifang Medical University, 2428 Yuhe Road, Weifang, 261042 China; 2 Department of Obstetrics and Gynaecology, Qilu Hospital, Shandong University, Jinan, China

**Keywords:** Human papillomavirus, Viral load, Cervical intraepithelial lesion, Influential factor

## Abstract

**Background:**

High-risk human papillomavirus (HR-HPV) load is thought to be influenced by many factors, and the relationship between viral load and the degree of cervical lesion is controversial. This study explored the possible influencing factors of HR-HPV viral load in the uterine cervix.

**Methods:**

A total of 605 women who needed colposcopic evaluation for abnormal cervical screening at the Affiliated Hospital of Weifang Medical University, China, between November 2017 and September 2018 were enrolled. Cervical specimens were collected from the endo- and ectocervix separately using two different cervical brushes. The hybrid capture II test was used to measure HR-HPV load. Age, histological severity, number of viral types, and area and location of cervical lesions were recorded. The correlations between viral load and influencing factors were analysed using univariate and multivariate analyses.

**Results:**

HR-HPV load was positively correlated with age, histological severity, multiple HPV types and area of cervical lesions (*P* < 0.05). Viral load with the combination of endo- and ectocervical sampling was significantly higher than simple endocervical sampling (*P* < 0.001). Multivariate analysis showed that age, multiple HPV types and area of cervical lesions were independent factors for HR-HPV load with a combination of endo- and ectocervical sampling (*P* < 0.05). However, only age and area of cervical lesions were independent factors for viral load with simple endocervical sampling (*P* < 0.05). No significant association was found between viral load and lesion severity in multivariate analysis (*P* > 0.05).

**Conclusion:**

HR-HPV load is influenced by age, histological severity, multiple viral types, area of cervical lesion and sampling methods. Age and area of cervical lesions are independent factors for viral load.

## Background

Cervical cancer is the second most common gynaecological cancer worldwide. It is well established that almost all precancerous and cancerous lesions of the cervix are caused by persistent oncogenic high-risk human papillomavirus (HR-HPV) infection. Therefore, HPV DNA testing was recently recommended as an alternative to cytology-based cervical cancer screening by the American updated screening guidelines [1]. However, it is less specific and has low positive predictive value, which is incapable of differentiating transient and persistent infections [2], resulting in excessive management of women with innocuous HPV infections [3]. Therefore, how to best triage HPV-positive women through secondary screening to identify those women with true precancerous lesions remains a pending issue in cervical cancer screening.

Many epidemiologic studies have provided strong and consistent evidence that persistent infection with HR-HPV is responsible for the development, maintenance and progression of cervical intraepithelial lesions (SILs) and cervical cancer (CC) [4, 5]. Persistence may depend on certain characteristics, such as high viral load as a result of viral replication [5]. However, the relation between HR-HPV viral load and future risk of HSIL or CC is less certain. Many studies have reported that viral load increases with the elevation of disease severity [6, 7], while others have not confirmed such an increased risk of disease progression [8]. High viral load has been found to be associated with the persistence of infection [5], which is an important event in HR-HPV-associated neoplastic progression [4, 5]. Therefore, a high viral load may also be associated with the elevation of disease severity. However, another important event in HPV-associated neoplastic progression is deregulation of normal patterns of virus gene expression [9, 10]. There appear to be multiple causes of deregulated HPV gene expression, and the most common cause in cervical cancer is integration of the virus genome into host chromosomes [10]. Integration of the viral genome, which disrupts the E1 and E2 genes and leads to increased expression of the E6 and E7 oncoproteins [10], results in viral life cycle termination and a reduction in the actual viral DNA copy number [11]. Furthermore, there are probably undetermined confounding factors influencing HR-HPV load [5, 12–14].

Based on the abovementioned findings, we aim to explore the possible influencing factors of HR-HPV viral load in the uterine cervix. The results of this study are expected to contribute to an understanding of the association of viral load and the severity of cervical lesions.

## Materials and methods

### Study population

**Fig. 1:**
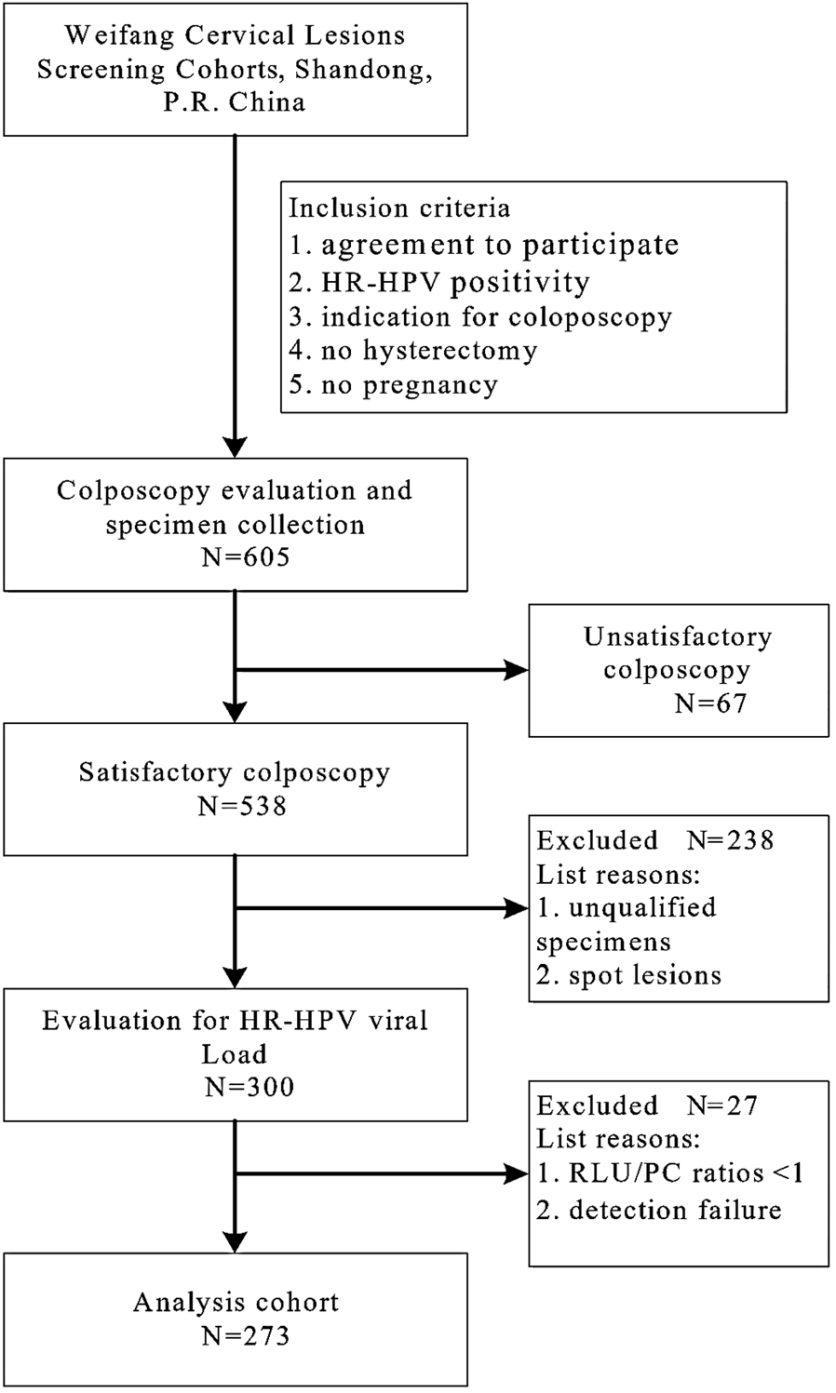
Flowchart of patient inclusion in the study. Unsatisfactory colposcopy, cervical lesions not completely visible. *RLU* relative light unit, *PC* positive control

All clinical samples in this study were organized using the Weifang Cervical Lesions Screening cohorts, Shandong, P. R. China. Detection of the 21 HPV genotypes was performed by the HPV GenoArray Test Kit (HybriBio Ltd., China) before enrolment of patients. A total of 605 women who needed colposcopic evaluation for abnormal cervical screening at the Affiliated Hospital of Weifang Medical University, China, between November 2017 and September 2018 were enrolled. The inclusion criteria were as follows: (1) agreement to participate; (2) HR-HPV positivity; (3) indication for colposcopy [15]; (4) no hysterectomy; and (5) no pregnancy. Women with cervical lesions that were not completely visible (unsatisfactory colposcopy) were excluded (Fig. 1). In total, 273 women were eligible for the study. All participants signed an informed consent document before enrolment. Women’s ages ranged from 21 to 63 years (38.4 ± 9.0 years). Based on the number of HPV types in primary screening, women were divided into the following two groups: multiple HPV types (at least two or more genotypes of HR-HPV infection) and single HPV type (only one genotype of HR-HPV infection).

### Specimens collection

At first, one physician (T. T. Wang) who was trained used a cytobrush (Digene Cervical Sampler, USA) to collect exfoliated cells from the endocervix (an area that can be covered by a cytobrush, bounded below by the ectocervix within a 0.5-cm radius of the external orifice of the uterus). Then, a different cervical brush (ThinPrep ® Cytologic Test Sampler, USA) was used to collect exfoliated cells of the ectocervix (an area that cannot be covered by a cytobrush bounded above by the ectocervix over a 0.5-cm radius of the external orifice of the uterus). Both specimens were separately transferred to specimen preserving medium (formalin), stored at − 4 °C and sent to the laboratory for viral load testing using the commercially available Hybrid Capture II system (Digene, USA). Based on the anatomical sites of sampling, sampling methods were stratified into two groups: a simple endocervical sampling group and a combination of endo- and ectocervical sampling group.

### Colposcopic examination

A standard colposcopic examination was performed by the same professional colposcopist (Y.Z. Liu). Cervical lesions showing aceto-white epithelium and/or atypical vessel changes (mosaic, punctation and tumour vessels) were measured under 15X magnification of colposcopy. The area (cm^2^) and location (the distance between the external orifice of the uterus and the below bound of the lesions, cm) of the lesions were quantified using a colposcopy image processor. Based on the location of lesions, women were stratified into two groups: involvement of the area bounded below by the ectocervix within a 0.5-cm radius of the external orifice of the uterus (≤ 0.5) and involvement of the area bounded below by the ectocervix over a 0.5-cm radius of the external orifice of the uterus (> 0.5). For pathology diagnosis, colposcopy with a directed biopsy was performed as indicated, and biopsy results were evaluated by two pathologists who were blind to the colposcopy results or clinical information. All cases with disagreement were reanalysed by another experienced pathologist. Based on colposcopy and pathology findings, the patients were stratified into three groups: negative (negative for intraepithelial lesion or malignancy), LSIL (low-grade squamous intraepithelial lesion), and HSIL/CC (high-grade squamous intraepithelial lesion and/or cervical cancer).

### Detection of HPV-DNA

HPV genotyping by HybriMax was performed using an HPV GenoArray Test Kit (HybriBio Ltd., China). This assay can determine 21 HPV types, including 14 high-risk HPV types (16, 18, 31, 33, 35, 39, 45, 51, 52, 56, 58, 59, 66, and 68), five low-risk HPV types (6, 11, 42, 43, and 44), and two unknown-risk types (53 and CP8304), by the flow-through hybridization technique using HPV DNA amplified by PCR as described before [16].

A hybrid capture II assay (Digene, USA) was performed on cervical scrapings collected from the endo- and ectocervix (bounded by the 0.5-cm radius of the external orifice of the uterus) separately using two different cervical brushes. Light measurements were quantified using a luminometer and were expressed by comparing the relative light units (RLU) of clinical samples with a positive control (PC). RLU/PC ratios (RLUs) were calculated as the ratio of the specimen luminescence to the luminescence of the 1.0 pg/mL HPV cut-off standard, which is known to represent a semiquantitative value for the cumulative viral burden of one or more of the 13 oncogenic HPV genotypes (types 16, 18, 31, 33, 35, 39, 45, 51, 52, 56, 58, 59 and 68). An RLU/PC ratio of 1 or higher was considered a positive result as proposed by the manufacturer (equivalent to 1 pg/mL of HPV-DNA).

### Statistical analysis

All statistical analyses were performed using SPSS version 22.0 for Windows. Means, medians, ranges, standard deviations, 95% confidence intervals, and standard errors of means were used as descriptive statistics. Viral load was measured as RLU/PC ratios (RLUs) and agreed with previous specifications in the hybrid capture assay. Viral quantification data in RLUs were initially continuous measurements. RLUs were transformed into their logarithm (Log10). An index of the Spearman correlation coefficient (*r*_s_) was applied to measure the association between viral load and variables. Multiple linear regression analysis was applied to relate the viral load with variables. The variables included in the model were age, histological severity, number of HPV types, area and location of the cervical lesions. A paired *t*-test was used to compare viral load with different sampling methods. Two-tailed *P* values less than 0.05 were considered statistically significant.

## Results

### Correlation of HR-HPV load and age

The median age of the 273 women was 39 years (range, 21–63 years). A significant association between viral load and age was found (*r*_sendocervical_ = 0.417, *P*_endocervical_ = 0.001, *r*_sendo-andectocervical_ = 0.349, *P*_endo-andectocervical_ = 0.001). A distinct upward trend of viral load paralleled increasing age (Table 1).

**Table 1:** Univariate analysis of factors associated with HR-HPV load

Variables	Parameters	Number of patients (%)	Simple endocervical sampling			Combination of endo-and ectocervical sampling		
			Mean (SD) of log_10_ transformed viral load	*r*_s_-value^a^	*P*-value	Mean (SD) of log_10_ transformed viral load	*r*_s_-value^a^	*P*-value
Age (years)	20–29	52 (19.0)	0.57 (± 0.62)	0.417	0.001	0.76 (± 0.61)	0.349	0.001
	30–39	91 (33.3)	0.92 (± 0.73)			1.11 (± 0.67)		
	40–49	99 (36.3)	1.16 (± 0.78)			1.29 (± 0.78)		
	50–59	25 (9.2)	1.41 (± 0.69)			1.63 (± 0.72)		
	60–69	6 (2.2)	2.52 (± 0.13)			2.56 (± 0.17)		
Lesion severity	Negative	90 (33.0)	0.79 (± 0.39)	0.371	0.002	0.88 (± 0.35)	0.424	< 0.001
	LSIL	93 (34.1)	0.87 (± 0.91)			1.03 (± 0.89)		
	HSIL/CC	90 (33.0)	1.41 (± 0.81)			1.66 (± 0.70)		
Multiple HPV types	No	200 (73.3)	0.92 (± 0.71)	0.198	0.060	1.08 (± 0.65)	0.237	0.024
	Yes	73 (26.7)	1.29 (± 0.93)			1.48 (± 0.95)		
Area of lesions (cm^2^)	0.0–0.49	174 (63.7)	0.95 (± 0.74)	0.292	0.017	1.04 (± 0.70)	0.364	< 0.001
	0.5–0.99	46 (16.8)	1.03 (± 0.72)			1.22 (± 0.69)		
	1.0–1.49	24 (8.8)	0.93 (± 0.82)			1.41 (± 0.75)		
	1.5–1.99	6 (2.2)	1.64 (± 0.94)			1.80 (± 0.77)		
	2.0–2.49	10 (3.7)	1.76 (± 0.86)			1.90 (± 0.73)		
	2.5–2.99	7 (2.6)	0.27 (± 0.14)			0.72 (± 0.01)		
	3.0–3.49	6 (2.2)	2.43 (± 0.41)			2.89 (± 0.06)		
Location of lesions (cm)	≤ 0.5	125 (68.3)	1.05 (± 0.72)	− 0.017	0.891	1.21 (± 0.65)	− 0.129	0.222
	> 0.5	58 (31.7)	0.90 (± 1.01)			1.10 (± 1.09)		

*Negative* negative for intraepithelial lesion or malignancy, *LSIL* low-grade squamous intraepithelial lesion, *HSIL/CC* high-grade squamous intraepithelial lesion and/or cervical cancer

^a^Spearman correlation coefficient. SD, standard deviation. Location of lesions, the distance between the external orifice of the uterus and the below bound of the lesions. ≤ 0.5 cm, involvement of the area bounded below by the ectocervix within a 0.5-cm radius of the external orifice of the uterus; > 0.5 cm, involvement of the area bounded below by the ectocervix over a 0.5-cm radius of the external orifice of the uterus. Multiple HPV types, at least two or more genotypes of HR-HPV infection

### Correlation of HR-HPV load and histological severity

Based on the colposcopy and pathology findings, 90 of the women were pathologically negative, and 93 were diagnosed with LSIL, 72 with HSIL and 18 with CC. In the simple endocervical sampling method, there were marked increases in log_10_-transformed viral load (log_10_RLUs) from the mean value of 0.79 (± 0.39) in the pathologically negative group to 0.87 (± 0.91) and 1.41 (± 0.81) in the LSIL and HSIL/CC groups, respectively (*r*_s_ = 0.371, *P* = 0.002). Similarly, in combination with endo- and ectocervical sampling methods, distinct upward trends of log_10_-transformed viral load (log_10_RLUs) were observed from a mean value of 0.88 (± 0.35) in the pathologically negative group to 1.03 (± 0.89) and 1.66 (± 0.70) in the LSIL and HSIL/CC groups, respectively (*r*_s_ = 0.424, *P* < 0.001) (Table 1).

### Correlation of HR-HPV load and multiple HPV types

In this population, 73.3% (200/273) of the women were positive for a single viral type. A total of 26.7% (73/273) were positive for multiple viral types. In the simple endocervical sampling method, no significant association between viral load and multiple HPV types was found (*r*_s_ = 0.198, *P* = 0.060). In the combination of endo- and ectocervical sampling methods, the multiple HPV type infection group showed a significantly higher viral load than the single HPV type infection group (*r*_s_ = 0.237, *P* = 0.024) (Table 1).

### Correlation of HR-HPV load and area of cervical lesions

Viral load was significantly associated with the area of the cervical lesions (*r*_sendocervical_ = 0.292, *P*_endocervical_ = 0.017, *r*_sendo-andectocervical_ = 0.364, *P*_endo-andectocervical_ < 0.001). A distinct upward trend of viral load paralleled the increasing area of cervical lesions (Table 1).

### Correlation of HR-HPV viral load and location of cervical lesions

Among the 183 women with colposcopy-detectable cervical lesions, there were 125 and 58 cases with lesions involving the area bounded below by the ectocervix within a 0.5-cm radius (≤ 0.5) and bounded below by the ectocervix over a 0.5-cm radius (> 0.5) of the external orifice of uterus, respectively. No statistical association was identified between viral load and location of the cervical lesions (*r*_sendocervical_ = − 0.017, *P*_endocervical_ = 0.891, *r*_sendo-andectocervical_ = − 0.129, *P*_endo-andectocervical_ = 0.222) (Table 1).

### Correlation of HR-HPV load and sampling methods

The value of log_10_-transformed viral load (log_10_RLUs) showed a significant difference between simple endocervical sampling and the combination of endo- and ectocervical sampling (1.19 ± 0.76 vs. 1.02 ± 0.790). Viral load with the combination of endo- and ectocervical sampling was significantly higher than with simple endocervical sampling (*t* = 9.67, *P* < 0.001) (Table 2).

**Table 2:** Comparison of HR-HPV load between simple endocervical sampling and combination of endo- and ectocervical sampling

Sampling method	Simple endocervical sampling	Combination of endo- and ectocervical sampling	95% CI	*t*-value^a^	*P*-value
Mean(SD) of log_10_ transformed viral load	1.02 (± 0.79)	1.19 (± 0.76)	0.130–0.197	9.67	< 0.001

*95% CI* 95% confidence interval

^a^Paired *t*-test

### Multivariate analysis for viral load

All the variables were included and were found to be significant according to univariate analysis in the present study. In simple endocervical sampling, this analysis showed that only age and area of cervical lesions were independent influencing factors for HR-HPV load (*P*_age_ = 0.001, *P*_areaoflesions_ = 0.010). In combination with endo- and ectocervical sampling methods, this analysis found that age, multiple HPV types, and area of cervical lesions were independent influencing factors for HR-HPV load (*P*_age_ = 0.004, *P*_multipleHPVtypes_ = 0.011, *P*_areaoflesions_ < 0.001) (Tables 3, 4).

**Table 3:** Multivariate analysis of factors associated with HR-HPV load in simple endocervical sampling

Variables	Unstandardized coefficients		Standardised coefficients	*t*-value^a^	*P*-value
	*β* value	*SE*	*β* value		
Age	4.695	1.316	0.376	3.567	0.001
Lesion severity	9.441	19.213	0.062	0.491	0.625
Area of lesions	46.615	17.525	0.339	2.660	0.010

*SE* standard error

^a^Multiple linear regression analysis

**Table 4:** Multivariate analysis of factors associated with HR-HPV load in combination of endo- and ectocervical sampling

Variables	Unstandardized coefficients		Standardised coefficients	*t*-value^a^	*P*-value
	*β* value	*SE*	*β* value		
Age	4.181	1.399	0.252	2.990	0.004
Lesion severity	10.878	18.841	0.059	0.577	0.565
Multiple HPV types	72.833	27.984	0.219	2.603	0.011
Area of lesions	96.290	20.641	0.480	4.665	< 0.001

*SE* standard error

^a^Multiple linear regression analysis

## Discussion

Persistent high-risk human papillomavirus (HR-HPV) infection is the main cause of precancerous and cancerous lesions of the cervix [4, 5]. Viral load reflects the number of infected cells and viral copies in individual cells. High viral load has been suggested as a marker of nontransient infection [5]. Therefore, a high viral load may also be associated with the progression of cervical lesions. However, the association between HPV viral load and cervical lesion grade is controversial. There could be undetermined confounding factors, such as age, number of viral types, area and location of cervical lesions [5, 12–14]. Few studies have incorporated these factors into the analysis, so the influence of confounding factors cannot be excluded. In the present study, we conducted a multiple linear regression analysis to evaluate factors associated with HR-HPV load. To analyse the influence of sampling factors on viral load, two different sampling methods were used to obtain cervical specimens for viral load detection. The aim of this study was to explore the possible influencing factors of viral load.

In this study, the results showed that age was an independent factor for HR-HPV load and that a significantly increasing age correlated with a higher viral load. This result agrees with previous population-based studies [17, 18], which showed that HR-HPV load was higher in older age. Different from previous studies, this study incorporated different variables into the analysis to adjust the effect of confounding factors and showed that age was an independent factor for HR-HPV load. However, contrary to our finding, Flores R et al. reported that HR-HPV load declined with age and was significantly higher in younger women [19]. They divided women into three age groups (15–24, 25–32, 32–79 years) and found that younger women presented higher viral load. This observation is understandable, and the effect of age on viral load is probably related to the balance between new acquisition of HR-HPV infections and viral clearance. Young women (< 25 years old) may represent new exposures to HPV due to sexual debut when an immune response to HPV has not yet been established. Therefore, the rate of new acquisition of HR-HPV infections exceeds the clearance rate [20], favouring the rapid accumulation of infections, which might contribute to a high viral load at an early age. Older women (> 45 years old) are more likely to experience physiological and immunological disorders during the menopausal transition. Such physiologic and immunologic dysregulation can result in an inability to establish an immune response to HPV, and a high level of HPV infections cannot achieve spontaneous clearance [13, 21].

Several studies have reported the association of high viral load with the risk for cervical cancer and its precursors. A large number of studies used the Hybrid Capture II assay to measure viral load, and while some found viral load to be positively associated with increased severity of cervical lesions [6, 7], others did not [8]. Different from previous studies, the present study explored the relationship between HR-HPV load and cervical lesion severity when other cofactors were taken into consideration. In agreement with some investigators [6, 7], this study showed a positive association between cervical lesion severity and viral load. However, this association lost significance when other cofactors, including age, presence of multiple HR-HPV infections and area of cervical lesions, were considered in multivariate analysis. This fact could be explained by the bias of Hybrid Capture II viral load. One major bias is that this test does not provide an evaluation of cell numbers, which vary substantially with different areas of cervical lesions. Moreover, high viral load detected by Hybrid Capture II may represent single or multiple HPV types among the 13 high-risk types detected by the kit, but this test does not provide the evaluation of the number of HR-HPV types. Therefore, these factors (area and presence of multiple HR-HPV infections) may confound the association between HR-HPV load and cervical lesion severity. Therefore, HR-HPV load determined by Hybrid Capture II alone may not be used as a molecular biomarker of risk for developing cervical (pre-) cancerous lesions.

The prevalence rate of concurrent multiple HPV genotype infections was reported to be approximately 20%-25% in several large studies [22, 23], which was nearly consistent with our result (26.7%). The role of multiple infections in carcinogenesis, with synergistic or antagonistic effects, remains to be determined [24, 25], although some studies have considered them to be a risk factor for HPV persistence and for preinvasive and invasive cervical lesions [24]. In this study, we analysed the correlation between multiple HPV genotype infections and HR-HPV load, which was calculated by two sampling methods, when possible confounding factors were incorporated into the analysis. The data indicated that multiple HPV genotype infections presented with significantly higher viral loads than single HPV genotype infections, but not significantly so in the simple endocervical sampling method, probably due to the impact of sampling variation on viral load and the relatively small-scale population. This finding indicates that cervical lesions induced by multiple HPV genotype infections are more likely to be associated with increased viral copies.

Current data showed that a distinct upward trend of viral load paralleled the increasing area of cervical lesions in both univariate and multivariate analyses. This result corroborates previous studies that have reported that more severe lesions tended to be larger and to harbour more HPV viral copies [26–28]. Different from previous studies, this study incorporated different variables into the analysis to adjust for the effects of confounding factors and showed that the area of cervical lesions is an independent factor determining viral load. This finding indicates that viral load is strongly affected by the number of infected cells, which increases in parallel with the increasing area of squamous intraepithelial lesions. However, to explore the association between HPV viral load and cervical lesion grade, some studies normalized viral load by using human cells to adjust the absolute viral load (e.g., expressed by copies/10,000 cells) to lessen the impact of the variation of sample volume [29]. This may lead to ignoring the effect of the number of infected cells on viral load.

HPV viral load estimated from cervical scrapings can be easily affected by sampling [14]. The radius of the cytobrush, such as a Digene cervical sampler, which is commonly used in HPV tests, is 0.5 cm. It is limited to collecting specimens in the area of the external orifice of the uterus within a 0.5-cm radius. When cervical lesions extend to the area of the external orifice of the uterus over a 0.5-cm radius, the brush cannot fully reach them, resulting in the collection of an inadequate number of cells that would underestimate the actual viral load. Therefore, to evaluate the impact of sampling methods on viral load, we compared the currently used simple endocervical sampling method with a new method of a combination of endo- and ectocervical sampling. We found that the viral load with a combination of endo- and ectocervical sampling was significantly higher than that with simple endocervical sampling. However, there was no significant difference in viral load between the lesions located in the area of the external orifice of the uterus within and over a 0.5-cm radius. This may be explained by a possible biased selection of participants in our study, in that only a minor proportion of the enrolled women with cervical lesions involved the area of the external orifice of the uterus over a 0.5-cm radius.

To our knowledge, our study is the first to demonstrate that age and area of cervical lesions are independent factors determining viral load, which may contribute valuable data for future discussions related to the potential application of viral load measurement. Nevertheless, several limitations also need to be addressed. First, the number of women with cervical lesions involving the area of the external orifice of the uterus over a 0.5-cm radius is too limited to draw a definitive conclusion for the effect of location of the cervical lesions on viral load and specimen collection methods. In addition, clinical data collected in one institution limit our statistical power to draw definitive conclusions for populations worldwide. Finally, the sample size in our study hinders our capacity to evaluate the association of HR-HPV load with other potential confounders, such as sampling physicians and individual HPV types. Further intensive clinical setup and laboratory investigations are therefore needed.

## Conclusion

High-risk human papillomavirus (HR-HPV) load is influenced by age, histological severity, multiple viral types, area of cervical lesions and sampling methods. Age and area of cervical lesions are independent factors for viral load. Comprehensive sampling is needed, especially when the cervical lesions are located far from the cervical orifice of the uterus.

## Data Availability

The data used to support the findings of this study are mainly included within the article, and the underlying data are available from the corresponding author upon request.

## Abbreviations

LSIL: Low-grade squamous intraepithelial lesion
HSIL: High-grade squamous intraepithelial lesion
CC: Cervical cancer
HR-HPV: High-risk-Human papillomavirus
HCII: Hybrid capture II
TCT: Thinprep cytological test
HPV-DNA: Human papillomavirus deoxyribonucleic acid
ASC-US: Atypical squamous cell of undetermined significance
RLU: Relative light units
PC: Positive control
